# The burden of knee pain in the operating room: are surgical personnel at greater risk?

**DOI:** 10.55730/1300-0144.6150

**Published:** 2026-01-22

**Authors:** Ömer Faruk NALDÖVEN, Yavuz KARAMAN, Başak Sinem SEZGİN, Şahan GÜVEN, Enejd VEİZİ, İzzet BİNGÖL

**Affiliations:** 1 Department of Orthopedics and Traumatology, Ankara Bilkent City Hospital, Ankara, Turkiye; 2 Department of Orthopedics and Traumatology, Ankara Yıldırım Beyazıt University, Ankara, Turkiye

**Keywords:** Knee pain, operating room, surgical personnel, work-related musculoskeletal disorders, surgical hand scrubbing

## Abstract

**Background/aim:**

This study aimed to determine the prevalence of knee pain among healthcare workers in surgical units and to explore whether the use of knee panels during surgical hand scrubbing is associated with persistent knee pain.

**Materials and methods:**

This single-center cross-sectional study was conducted between October 2023 and March 2025. A total of 400 physicians and nurses working in surgical and nonsurgical departments were included. Participants completed a structured and modified Nordic Musculoskeletal Questionnaire, as well as knee pain assessment tools, including a visual analog scale (VAS) and the Kujala score for anterior knee pain-related symptoms. Occupational exposure among surgical staff was additionally evaluated. Data were analyzed using IBM SPSS Statistics.

**Results:**

The prevalence of knee pain was 54.8% among surgical personnel and 39.5% among nonsurgical personnel. VAS scores were significantly higher in the surgical group, while there was no significant difference between groups in terms of Kujala scores. Knee panel use was common and more than half of the users reported discomfort during use; however, there was no statistically significant association between panel use and persistent knee pain. Regression analysis identified increasing age, longer duration of surgical experience, and employment in a surgical unit as factors independently associated with knee pain.

**Conclusion:**

Employment in a surgical unit is associated with a higher prevalence of knee pain among healthcare workers. However, the use of knee panels during surgical hand scrubbing does not appear to be associated with persistent knee pain. Multicomponent ergonomic interventions should be implemented to prevent musculoskeletal disorders.

## 1. Introduction

Work-related musculoskeletal disorders (WMSDs) are among the most common occupational health problems worldwide [1]. These conditions lead to a decline in quality of life on an individual level while also contributing to workforce loss and service disruption within healthcare systems [2]. WMSDs include both specific diagnoses, such as tendinopathies, nerve entrapments, and joint or capsule disorders, and nonspecific musculoskeletal complaints characterized by chronic pain or tenderness without clear pathological findings [3].

Healthcare workers are considered a high-risk group for WMSDs due to psychosocial stressors and demanding work conditions. Among them, operating room nurses [4] and surgeons [5] are especially vulnerable, primarily due to prolonged standing, repetitive tasks, and poor ergonomic postures during procedures [6].

While WMSDs often affect multiple body regions, anterior knee pain is frequently reported [7]. One of the most common causes of anterior knee pain is patellofemoral pain, a multifactorial condition associated with repetitive microtrauma and increased metabolic activity in the patellofemoral joint [8]. Activities involving high patellar load, such as squatting, stair climbing, and knee bending, can exacerbate symptoms [9,10]. An emerging body of literature suggests that patellofemoral pain is not solely related to structural or anatomical abnormalities but also involves mechanical stress, impaired tissue homeostasis, and localized inflammatory processes [11,12].

To maintain surgical hygiene, knee-activated panels are frequently used by operating room staff during preoperative scrubbing. However, repeated pressure on these panels may impose mechanical stress on the same area of the patellofemoral joint, potentially contributing to pain development. Such exposure has been hypothesized to cause transient discomfort; however, its relationship with persistent knee pain remains unclear. Despite this plausible link, the impact of knee panel use on persistent knee pain has not been previously examined in the literature.

This study aims to evaluate the relationship between working in surgical units and the presence of knee pain and to investigate whether the use of knee panels during surgical handwashing is associated with persistent anterior knee pain. By identifying occupational risk factors, we aim to contribute to the development of ergonomic strategies that promote musculoskeletal health among healthcare workers.

## 2. Materials and methods

### 2.1. Study design and setting

This single-center cross-sectional study was conducted between October 2023 and March 2025 at Ankara Bilkent City Hospital, targeting physicians and nurses working in internal medicine and surgical departments. The study commenced following approval from the local ethics committee (Approval No: E1-23-3718, Date: 06.09.2023), and written informed consent was obtained from all participants. Participation was voluntary, all personal data were kept confidential, and individuals had the right to decline or withdraw from the study at any stage.

The study population included physicians and nurses aged 18–50 years who had been actively working in either internal medicine or surgical units for at least 2 years. Exclusion criteria included the presence of systemic, rheumatologic, or psychiatric diseases; a known history of knee pathology or previous knee surgery; use of medications with known musculoskeletal side effects; and incomplete data.

### 2.2. Instruments and outcome measures

Participants completed a structured four-part questionnaire that took approximately 10 min to complete. The questionnaire was developed after reviewing the relevant literature and was based on a modified version of the Nordic Musculoskeletal Questionnaire [13], with a specific focus on the knee region. Additional validated tools were incorporated to enhance clinical evaluation, including a visual analog scale (VAS) and the Kujala score for anterior knee pain-related symptoms[14].

The questionnaire consisted of the following sections:

#### i.Demographic and Professional Information

Age, sex, height, weight, body mass index (BMI), profession (physician/nurse), current department (surgical/internal), years of professional experience, history of systemic illness, regular medication use, and prior surgical history.

#### ii. Knee Pain History

Presence of any knee pain or discomfort within the last 12 months, duration of symptoms, severity of pain assessed using a 0–10 VAS, and whether the participant had ever sought outpatient care for these complaints

#### iii. Surgery-Specific Occupational Exposure

This section was administered only to participants in surgical departments. It included questions about years of experience in surgical procedures, average number of surgeries per week, use of knee panels during surgical scrubbing, and the presence of knee discomfort during panel use.

#### iv. Anterior Knee Pain Assessment

All participants completed the Kujala score using a validated questionnaire to assess symptoms and functional limitations related to anterior knee pain.

### 2.3. Data analysis

All statistical analyses were conducted using IBM SPSS Statistics 25.0 for Windows (IBM Corp., Armonk, NY, USA). Descriptive statistics were presented as mean ± standard deviation for continuous variables and as frequency (n) and percentage (%) for categorical variables.

The normality of the data distribution was assessed using the Kolmogorov–Smirnov test. For comparisons between the surgical and nonsurgical groups, an independent-samples t-test or the Mann–Whitney U test was applied for continuous variables, depending on the distribution. The chi-square test was used for categorical variables.

Univariate logistic regression analysis was initially performed to evaluate the associations between potential risk factors and the presence of knee pain. Variables with values of p < 0.20 in univariate analysis were included in the multivariate logistic regression model to identify independent predictors. Odds ratios (ORs) and 95% confidence intervals (CIs) were reported. Values of p < 0.05 were considered statistically significant.

## 3. Results

The demographic and occupational characteristics of the study population are presented in Table 1. A total of 400 healthcare workers participated in the study. The mean age of participants was 33.6 ± 7.5 years, ranging from 23 to 50 years.

Regarding sex distribution, 164 participants (41.0%) were male and 236 (59.0%) were female. The mean BMI was 25.2 ± 4.5, with values ranging from 16.9 to 43.5.

In terms of job roles, 48 participants (12.0%) were ward physicians, 144 (36.0%) were surgeons, 104 (26.0%) were surgical nurses, and 104 (26.0%) were ward nurses. Accordingly, 248 participants (62.0%) were classified as surgical staff, while 152 (38.0%) were nonsurgical staff.

Knee pain was reported by 136 participants (54.8%) in the surgical group compared to 60 participants (39.5%) in the nonsurgical group. This difference was statistically significant (p = 0.003). However, there was no difference between the groups regarding outpatient clinic visits for knee pain (Table 2).

Pain severity as measured by the VAS was significantly higher in the surgical group (mean: 2.6 ± 2.3; median: 3.0) compared to the nonsurgical group (mean: 2.2 ± 2.7; median: 0.0) (p = 0.023) (Table 2).

The Kujala score for anterior knee pain-related symptoms, used to assess anterior knee pain, was similar between groups: 87.5 ± 13.0 in the surgical group and 88.3 ± 10.4 in the nonsurgical group. The median score was 92.0 in both groups, with no statistically significant difference (p = 0.488) (Table 2).

**Figure 1:**
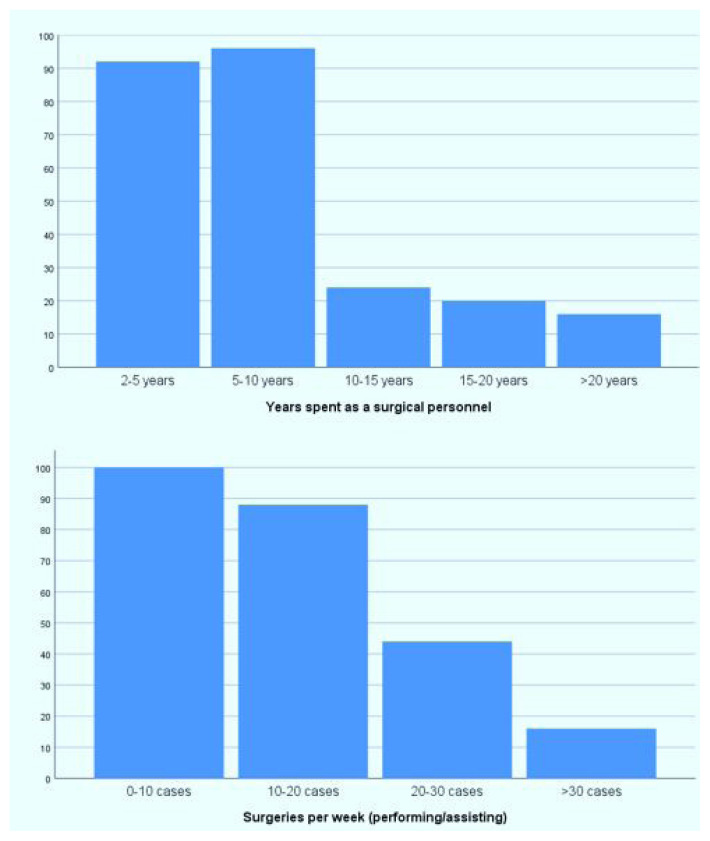
Distribution of surgical personnel by years of professional experience and weekly number of surgeries attended.

When evaluating years of professional experience among surgical staff, the highest proportion was observed in the group with 5–10 years of experience (n ≈ 95), followed by those with 2–5 years of experience (n ≈ 90). The number of participants with longer experience was lower (Figure 1). In terms of weekly surgical participation, the majority of surgical staff reported 0–10 and 10–20 cases per week, indicating a generally low to moderate surgical workload (Figure 1).

**Figure 2:**
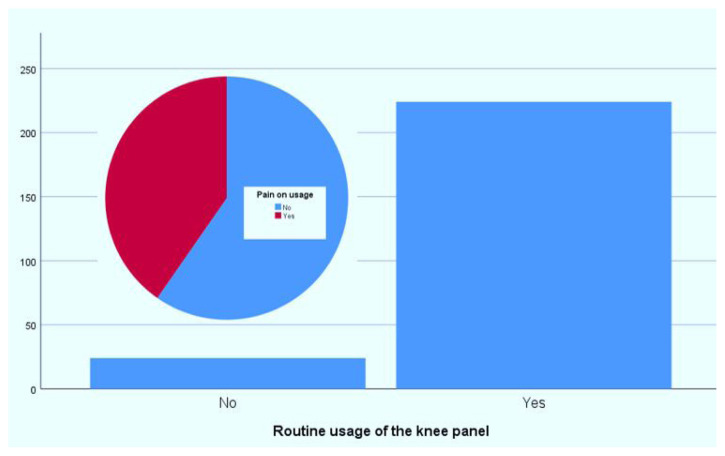
Routine use of the knee panel and presence of pain during usage among surgical staff.

Regarding the use of knee panels during surgical hand scrubbing, the vast majority of participants (n ≈ 230) reported using them regularly, while a smaller number (n ≈ 30) did not use knee panels (Figure 2). Among those who used panels, more than half reported knee discomfort during use (Figure 2), suggesting that knee panel use may cause discomfort in some individuals.

The results of univariate and multivariate logistic regression analyses for variables associated with the presence of knee pain are presented in Table 3. In univariate analysis, increasing age was significantly associated with a higher likelihood of knee pain. Similarly, a longer duration of surgical experience was associated with increased risk. Additionally, group classification (surgical vs. nonsurgical) showed a significant association with knee pain. However, no significant associations were found for sex, weekly surgery count, or knee panel use. In multivariate analysis, age and working in a surgical department were independently associated with knee pain.

## 5. Discussion

The most important finding of this study is that the prevalence of knee pain was significantly higher among surgical personnel compared to nonsurgical staff. Although the use of knee panels in the operating room environment was common, no significant correlation was observed between panel usage and persistent knee pain. These findings indicate an association between working in surgical settings and a higher prevalence of knee pain, potentially reflecting cumulative occupational and age-related factors. Nevertheless, using knee panels during surgical scrubbing was not identified as a contributing factor for persistent knee pain.

Operating room nurses and surgeons are known to be at higher risk of WMSDs due to both static and dynamic physical and biomechanical stressors [6,15]. Working in the operating room involves additional risk factors such as prolonged standing, awkward postures during surgeries, and rapid movements in emergency situations, which may contribute to the development of musculoskeletal disorders. These stressors can result in continuous static load and postural strain for this professional group. Dynamic pressures may stem from tasks involving pushing, pulling, or lifting heavy surgical equipment [16].

Previous studies have demonstrated the increased risk of WMSDs among surgical staff [5,6]. However, most of these studies lacked a control group for comparison. To address this gap, we compared surgical personnel with nonsurgical staff within the same institution, thereby providing a more controlled comparative perspective and highlighting occupation-related differences in knee pain prevalence.

In the literature on WMSDs, the most frequently affected body regions among surgical personnel include the back, lower back, and knees [4,15]. Our study focused specifically on knee disorders using a modified questionnaire and evaluated factors that may contribute to knee pain. The high prevalence of knee pain among operating room nurses and surgeons found in our study is consistent with previous findings in similar occupational groups.

During preoperative hand scrubbing, various water activation mechanisms such as knee panels, foot pedals, and hand sensors are used to enhance hygiene and prevent hand contact. Knee panels in particular may apply repeated mechanical stress to the patellofemoral joint. In our study, more than half of the participants who used knee panels reported discomfort during use. However, this discomfort was not statistically associated with persistent knee pain. This null finding suggests that while knee panel use may cause transient symptoms, it may not be sufficient alone to explain long-term knee pain among surgical personnel. Nonetheless, future prospective studies incorporating objective clinical assessments and imaging modalities may help clarify any potential long-term structural effects.

A major strength of this study is its comparative design, including both surgical and nonsurgical healthcare professionals, and its focus on an understudied potential risk factor: the use of knee panels during surgical hand scrubbing. Data were collected using a systematic structured questionnaire and validated measurement tools. However, there are limitations. The cross-sectional design does not allow for the establishment of causal relationships. Future longitudinal cohort studies are needed to better assess causality. Additionally, the evaluation of knee pain was based on self-reported data, without objective clinical or imaging confirmation, and potential confounding factors such as physical activity level, footwear, and nonoccupational knee loading could not be evaluated. These factors should be considered in future research.

In conclusion, employment in a surgical unit is associated with a higher prevalence of knee pain among healthcare professionals; however, the use of knee panels during surgical hand scrubbing does not appear to be associated with persistent knee pain. Multicomponent ergonomic interventions should be implemented to prevent WMSDs.

## Figures and Tables

**Table 1:** Baseline demographic data of the study cohort.

	All patients (n = 400)

**Age**	
Mean ± SD	33.6 ± 7.5
Median (min – max)	31 (23–50)

**Sex**	
Man	164 (41.0%)
Woman	236 (59.0%)

**BMI**	
Mean ± SD	25.2 ± 4.5
Median (min – max)	24.7 (16.9–43.5)

**Main Group**	
Ward Doctor	48 (12.0%)
Surgeon	144 (36.0%)
Surgical nurse	104 (26.0%)
Ward Nurse	104 (26.0%)

**Surgical Team**	
Nonsurgical	152 (38.0%)
Surgical	248 (62.0%)

**Table 2:** Knee pain incidence and relevant scores of the nonsurgical and surgical personnel.

	Nonsurgical personnel (n = 152)	Surgical personnel (n = 248)	p-value

**Knee pain**			
Yes	60 (39.5%)	136 (54.8%)	**0.003**
No	92 (60.5%)	112 (45.2%)	

**Outpatients visit for knee pain**			
Yes	36 (23.7%)	68 (27.4%)	0.408
No	116 (76.3%)	180 (72.6%)	

**VAS**			
Mean ± SD	2.2 ± 2.7	2.6 ± 2.3	**0.023**
Median (min–max)	0.0 (0.0–7.0)	3.0 (0.0–8.0)	

**Kujala Score**			
Mean ± SD	88.3 ± 10.4	87.5 ± 13.0	0.488
Median (min–max)	92.0 (63.0–100.0)	92.0 (41.0–100.0)	

**Table 3:** Regression analysis of the study variables with respect to the presence of knee pain.

	Presence of knee pain			
	Univariate Regression		Multivariate Regression	
	OR (95% CI)	p-value	OR (95% CI)	p-value
Age	**1.056(1.027**–**1.087)**	**<0.001**	**1.060(1.030**–**1.091)**	**<0.001**
Sex	1.415(0.948–2.111)	0.090		
Subgroup	**0.537(0.356**–**0.810)**	**0.003**	**2.010(1.316**–**3.070)**	**0.001**
Surgical years	**1.372(1.089**–**1.727)**	**0.007**		
Weekly cases	0.946(0.719–1.244)	0.691		
Usage of knee panel	1.733(0.713–4.214)	0.225		
